# Are verbatim transcripts necessary in applied qualitative research: experiences from two community-based intervention trials in Ghana

**DOI:** 10.1186/s12982-022-00115-w

**Published:** 2022-06-28

**Authors:** Zelee Hill, Charlotte Tawiah-Agyemang, Betty Kirkwood, Carl Kendall

**Affiliations:** 1 Institute for Global Health, University College London, 30 Guilford St., London, WC1N 1EH UK; 2 Kintampo Health Research Centre, P.O. Box 200, Kintampo, Ghana; 3 London School of Hygiene & Tropical Medicine, Keppel Street, London, WC1E 7HT UK; 4 Department of Global Community Health and Behavioral Sciences, Tulane University School of Public Health and Tropical Medicine, 1440 Canal Street, Suite 2350, New Orleans, LA 70112 USA

**Keywords:** Qualitative research, Methodology, Transcription, Field notes, Maternal and child health, Africa, Ghana

## Abstract

Conducting qualitative research within public health trials requires balancing timely data collection with the need to maintain data quality. Verbatim transcription of interviews is the conventional way of recording qualitative data, but is time consuming and can severely delay the availability of research findings. Expanding field notes into fair notes is a quicker alternative method, but is not usually recommended as interviewers select and interpret what they record. We used the fair note methodology in Ghana, and found that where research questions are relatively simple, and interviewers undergo sufficient training and supervision, fair notes can decrease data collection and analysis time, while still providing detailed and relevant information to the study team. Interviewers liked the method and felt it made them more reflective and analytical and improved their interview technique. The exception was focus group discussions, where the fair note approach failed to capture the interaction and richness of discussions, capturing group consensus rather than the discussions leading to this consensus.

## Background

The value of qualitative research within public health trials and programmes is increasingly recognized [1, 2], as demonstrated by its prominence in the United Kingdom Medical Research Council framework for the development and evaluation of complex interventions [3]. When done well qualitative research can improve the design, conduct, interpretation and transferability of intervention trials [4]. This is most likely to happen when qualitative research is integral to the trial rather than peripheral or an add on [2].

Conducting integrated qualitative research within intervention trials requires balancing the need to make findings available to the team in a timely manner, with the need to maintain data quality. One of the most time consuming components of qualitative research is the transcription of interviews. Verbatim transcription, a word for word reproduction of the interview, is the convention, and is considered to enhance the rigour and accuracy of the data [5, 6], but can severely delay the availability of research findings [7].

This paper highlights the lessons learnt in two large scale trials, conducted at the Kintampo Health Research Centre, Ghana, using expanded field notes, fair notes [8], to record data rather than verbatim transcription. Fair notes save time and capture the main topics of interviews, but are considered less accurate [7, 9]. During the two trials we learned valuable lessons about enhancing the quality of the fair note method. This paper outlines the rational for choosing fair notes rather than verbatim transcription, and our experiences using the method.

## Main text

The trials were conducted between 2000 and 2010. The Obaapvita trial tested the impact of Vitamin A supplementation on maternal mortality [10]; and the Newborn Home Intervention Trial (the Newhints trial) tested the impact of home visits by community health workers on neonatal mortality [11]. Within these trials, qualitative research was conducted prior to the trial to inform intervention design and data collection plans, and during the trial to identify emerging implementation issues, and to conduct process evaluations to understand the reasons why the interventions were or were not successful. In addition, specific sub studies were conducted exploring issues such as informal abortions and women’s understanding of being in a trial. This qualitative work included in-depth interviews, focus group discussions and trials of improved practice and resulted in eleven peer-reviewed articles [12–23]. The core qualitative data collection methods are shown in Table 1. In many cases these qualitative data were complemented by quantitative data.

**Table 1:** Description of the qualitative methods used in the trials

Trial name and aims	Aims of qualitative data collection	Year of study	Methods used (IDI-in depth interview FGD—focus group discussion)	Data collection team	Duration and average interviews per day
ObaapaVitA trial to assess the effect of weekly vitamin A supplementation on maternal mortality	Formative research to inform trial design Topics included: – Factors affecting adoption and adherence to capsules – Communication and distribution channels	2000	50 IDI and 6 FGD with women of reproductive age 30 IDI with husbands 13 IDI with drug sellers, birth attendants and health workers	3 interviewers and 2 senior social scientists	2 months, with 2 interviews a fieldworker per day
	Qualitative interviews to explore implementation issues Topics included: – Perceptions of the trial and the capsules – Exploration of specific implementation issues	2002–2008	84 FGD discussion with women of reproductive age	4 interviewers and 2 senior social scientists	Throughout the trial with 1 FGD per month
	Qualitative interviews to inform trial closure plans Topics included: – Questions and concerns about the trial ending	2008	4 FGD with trial fieldworkers and 4 with community members	3 interviewers and 1 senior social scientist	2 months with 1 FGD per week
Newhints trial to test the impact of home visits by community health workers on neonatal mortality	Formative research to inform trial design Topics included: – Gaps in the knowledge and practice of neonatal care – Barriers and facilitators to behaviour change – Current role of community health workers and their potential to deliver the intervention	2006–2007	25 birth narratives with recently delivered women 30 IDI and 2 FGDs with recently delivered or pregnant women 20 IDIs and 6 FGDs with birth attendants and grandmothers 12 IDIs and 2 FGDs with husbands 16 IDI with community health workers and 6 with supervisors Trials of improved practice with 5 recently delivered women	5 interviewers and 1 senior social scientist	2 months, with 1.5 interviews a fieldworker per day
	Process evaluation to understands reasons why the intervention was or was not successful Topics included: – Issues affecting coverage – Information provided during home visits – Barriers and facilitators to behaviour change – Acceptability of the intervention	2010	64 IDIs with women enrolled in Newhints 23 IDIs with community CHWs 15 IDIs with Health workers 20 IDIs with traditional birth attendants	3 interviewers and 1 senior social scientists	2 months with 3 interviews a fieldworker per day. Note interviews were relatively short

## Rationale for using fair notes

When we planned the formative research to design the communication strategy to maximise compliance with weekly vitamin A/placebo capsules in the ObaapaVitA trial, we were faced with a decision about how to record interview data. We had three choices:

- Audio record and take field notes during the interview, and use the audio recording to produce verbatim transcripts and the field notes to add non verbal communication and observations.
- Audio record and take field notes during the interview, and use the recording to expand the field notes into fair notes.
- Take field notes during the interview and expand on these into fair notes from memory [7].

With the advent of portable audio recording equipment verbatim transcription had replaced fair notes as the convention [24], and audio recording was advised if a fair note approach was used [9]. However, the time demands of transcription were problematic for the ObaapaVitA qualitative research team, who needed to deliver usable data to the trial team within a few months. The simplicity and rapidity of fair notes was attractive, so the team compared these data recording methods according to seven criteria as shown in Table 2.

**Table 2:** Comparison of different data recording methods

	Verbatim transcription	Field notes expanded from audio recording	Field notes expanded from memory
Time and cost [7, 9, 24, 26–28]	Time consuming and costly. 1 h of interview can take 6–10 h to transcribe verbatim. The slow process can disrupt iteration Inclusion of repetitive or irrelevant discussions increases analysis time Hiring proficient typists may increase costs	Relatively quick. 1 h of interview can take 2–3 h to write up	Quicker than both the other methods
Completeness [5–7, 27–36]	Most complete method, although there may be equipment failures, poor equipment placement, environmental distractions, inaudible participants, deliberate alterations and transcription errors	Not complete, but checking the audio recording means less content is missed	Not complete, and no ability to check for missing content Content may be recalled incorrectly
Influence of interviewer/transcribers views [24, 27–29, 31, 33, 35–41]	Aim is to capture respondents’ words and non-verbal cues accurately. In reality transcripts are representations of the interview and are selective, subjective and interpretive	Exact words are not always captured and the write up represents the interviewers perspective. It can be very selective, subjective and interpretive. This can lead to simplistic or interview centric interpretations	Has the greatest potential for being selective, subjective and interpretive and to lead to simplistic interpretations
Quality assurance [5–7, 24, 35]	Re-listening to the audio recording for clarification and spot checking is possible, although spot checking is rarely done in studies	Re-listening to the audio recording for clarification and spot checking is possible	None
Other quality issues [27, 34, 42]	Can be tiresome for the transcriber, leading to a loss of enthusiasm and can impact quality of transcript Too much irrelevant detail may obscure key interview content during analysis Interviewers, knowing that the audio recorder is capturing the interview may tune out, and miss important leads for managing the interview		Requires the most skill on the part of the interviewers
Who does it [5, 7, 24, 39, 40, 42]	Transcription process facilitates knowledge, understanding and interpretation of the data, and is best done by the interviewer. Due to time issues it is often contracted out. This introduces an additional interpretation of the data into the analysis Participants may feel nervous being recorded	Done by the interviewers as soon after the interview as possible, allows the recording of ideas that would otherwise be lost	Done by the interviewers as soon after the interview as possible, allows the recording of ideas that would otherwise be lost
When is it best used [5–7, 9, 39]	Studies focusing on dialogue and language; cultural themes, or making detailed comparisons between segments of the population Some researchers believe that transcription should always be used	Studies focusing on thematic or content analysis that do not require a high degree of closeness to the data; or that aim to get rapid feedback from target populations to make programmatic decisions	Studies with simple research questions Where participants’ may be inhibited by being recorded

Table 2 shows that, although verbatim transcription is not an error free objective replication of the interview, it is the most complete method of recording data. It captures the respondent’s language most accurately, and permits quality assurance for the content of the interview. However, the ObapaVitA team calculated that if transcription was conducted by the interviewers, it would almost triple the duration of the formative research, especially as the interviewers were not expert typists. This meant that contracting transcription out would be the only feasible option to ensure that the formative research was compatible with the trial timeline. As shown in Table 2 contracting transcription out is problematic because it introduces an additional potential source of error into the analysis. It also means the research team loses control of the transcription process, and the team does not benefit from the analytical thinking that occurs during note-taking and transcription.

The ObaapaVitA team opted for the fair notes approach. This was mainly driven by time constraints, our relatively simple research questions, and a desire to keep data recording within the field team to enhance reflection and analytical thinking. Having decided on a field note approach we were faced with a choice of audio recording the interviews and using the audio recordings to expand the interviews, or using memory to expand the interviews. At the time the recording equipment available for the study was bulky, and the population we were working with was not used to the equipment. We were worried that being recorded would make participants feel nervous and inhibited. Although we knew that audio recording was recommended [9] we decided not to record the interviews.

Given the problems with expanding notes from memory that are outlined in Table 2, we planned for intensive training and supervision during data collection to help ensure important content was not lost, that the interviewers recorded the language of the participant as much as possible, and to aid in reflective and analytical thinking. The next section describes our experiences and lessons learnt in implementing this approach first describing our experience using the approach with interviews in the ObaapaVitA trial, the adaptation of the approach for the Newhints trial, and finally our experience using the approach for recording focus group discussions.

## Use of fair notes in the ObaapaVitA trial

Data were collected by five interviewers across the different rounds of qualitative research. During a 1 week training on qualitative methods and on the study we discussed writing up using the fair notes method. This component of the training focused on:

- The importance of the research for the trial and of the trial itself. This was included to motivate the interviewers to write detailed fair notes.
- The intent of each question on the interview guides, so that interviewers understood why the question was being asked and would be able to identify relevant interview content to include in their notes.
- The importance of capturing the voice of the participants rather than their own voice, but that fair notes should say ‘she said that she went to the shop…’ rather than ‘I went to the shop’ to be clear that it is not a verbatim transcript.
- The importance of interviewers recording their own thought and reflections, but that these should be clearly recorded in the fair notes using brackets [….].

The qualitative team then conducted several practice interviews, initially with each other, then with staff at Kintampo Health Research Centre and finally in the community. The senior project researchers leading the training workshops observed the practice interviews. Interviewers completed practice fair notes and discussed the notes line by line with a senior researcher. The interviewers then added to or changed their notes based on the discussion. The research team then met as a whole to review key lessons learnt about note taking and key findings from the interviews. During data collection we continued with one to one feedback and group discussions.

### Lessons learnt using fair notes in the ObaapaVitA trial

The practice interviews and write-ups identified three key problems with the fair note approach: taking detailed notes during the interviews, ‘tidying up’ fair notes and writing up brief fair notes.

Interviewers initially took very detailed notes during the interview, as they were fearful of forgetting things. This impacted on rapport building with the participant and meant that interviews were overlong. Senior researchers worked with the interviewers to help them trust their memories and interviewers practised taking concise, less obtrusive notes. Interviewers found that if they wrote up their interviews as soon as they returned to the field office, between 1 and 3 h after interviews were completed, they could easily remember what was discussed. This enhanced their confidence, reduced the length of the notes they took during the interviews and also increased the speed at which they wrote up their interviews. We developed a pattern of going to the field sites (1–2 h drive) early in the morning, conducting on average two interviews a day per fieldworker and then returning to the office to immediately start the write up which usually took the rest of the day. It was logistically easier to conduct two interviews a day, but this meant that interviewers had to rely more on their notes when converting the second interview into fair notes, whilst the write up of the first interview was always started between 1 and 3 h of data collection.

Interviewers wanted to make their fair notes read well, it was common for them to use technical terms that we knew were rarely used in the community. Whenever we saw such terms we used it as an opportunity to discuss ‘capturing the voice’ of the participant, for example we discussed the term ‘high blood pressure’, and found that the respondent had actually said ‘blood was up’. Through these discussions interviewers learnt that we were interested not just in what the participants said, but in capturing the words they used to say it.

Initially, despite detailed field notes, the fair notes were relatively brief summaries of the interviews, sometimes even bullet points extracted from more complete field notes taken during the interview. It took time for the interviewers to determine what a good fair note should consist of, this came through discussion and by asking interviewers to read each others write-ups. Discussing the intent of each question, and reviewing and discussing the fair notes, helped the interviewers understand the content that we were interested in. For example, we would read the fair notes and discuss why something was important for the study, and whether the respondent had said any more about the issue. Additional information would be added to the notes. We found that over one to two weeks the interviewers became aware of important content and the fair notes became longer and more detailed. We also found that the interviewers began to feel less like interviewers applying an interview guide and more like investigators—this enhanced the quality of the data in that interviewers probed more and were more likely to ask follow up questions on key issues, this was an unanticipated advantage of the approach. Interviewers who had previously transcribed interviews, reported that writing fair notes made them more reflective about their interviewing style, biases and perceptions and made them think analytically. They reported that this made writing up more enjoyable and enhanced their interviewing skills, they felt this increased their future employability and motivated them to do a good job.

It was important that interviews were conducted, written up, reviewed and corrected the same day when memories were fresh. Initially the process was quite slow, but as confidence grew and feedback reduced in length the process sped up, and interviewers were conducting and writing up an average of two interviews a day. The time it took to get to this stage varied by data collector but on average it took around two weeks. Keeping the interviewers motivated to complete quality write-ups was important. Senior researchers reviewing fair notes and going to the field with the interviewers showed that they cared about the study, and discussing findings made their use and importance clear. Interestingly interviewer motivation dipped at the same time data saturation was reached, as the interviewers complained that they were not learning new things and started to lose interest, this did not affect the quality of the fair notes, but interviews became shorter with fewer probes which was addressed through the feedback loop. Using the fair notes method, the research took 8 weeks of high intensity work, with an additional week of training. When the team reflected on the experience we felt that collecting data intensely over a short period of time enabled the interviewers to become immersed in the topic and maintain interest compared to our previous experience with transcription.

### Adaptation of the approach for the Newhints trial

From our experiences with the ObaapaVitA trial we were satisfied that, given the type of qualitative research questions asked within a trial, we could get useful data using the fair note approach. Small and compact recording devices had now become available to the study team, and the study population had become used to devices such as mobile phones. Given these changes we decided that we should maintain the fair notes approach, but audio record the interviews to allow interviewers to check for missed content, check language, to add key verbatim quotes and to allow for quality checks.

Based on one of the senior researcher’s experience with using audio recordings to expand field notes (ZH), we encouraged interviewers to first write fair notes from memory and then listen to the recording to check for completeness and to add key quotes-this was a quick process for the experienced interviewers with one hour of interview taking 1.5–2 h to write up. In practice most of the interviewers found it difficult not to rely on the audio recording, and despite several discussions almost all the interviewers listened to the recording and wrote up their notes as they went, this was a slow process and meant that interviewers could conduct and write up only one interview every day and a half. Data collection and analysis for the formative component of Newhints trial took longer per interviewer than for the ObaapaVitA trial. It also meant that interviewers were less likely to complete their write up as soon as they returned to the office as they knew they had the audio recordings to rely on at a later date. This meant that, at times, interviews were written up several days after the data were collected, which inhibited iterative data collection, interviewer reflection and disrupted the flow of the data collection-write up cycle.

### Using fair notes to record focus groups discussions

Both trials used focus group discussions as one of the data collection methods. Interviewers found these difficult to write up using the fair note method. They tended to record group consensus rather than the discussions that led to consensus being reached. We did not learn much from this data collection method, as the data were too summarized and not at all rich compared to the in-depth interview data. From the formative research for the ObaapaVitA trial, we realised that fair notes were not a good way of capturing focus group discussion data. For subsequent focus groups and for the Newhints trial all focus groups discussions were audio recorded and transcribed verbatim. Focus groups were a much richer and a more useful data source in this study, compared with in the ObaapaVitA trial, as the content and nuances of discussions were captured.

## Conclusion

This paper adds to the few papers that provide practical advice on fair notes and transcription within qualitative research [5]. We found, as have others, that where research questions are relatively simple, and interviewers undergo sufficient training and supervision, fair notes can decrease data collection and analysis time [7, 9]. Fair notes have been criticised for resulting in simplistic interpretations that underreport the participants’ words [7, 8], but we found that with training and supervision they can provide detailed and relevant information to the study team, and can enhance the quality of interviews and analysis—which has not been previously reported. The exception was data collected through focus group discussions, which was very difficult to write up using the fair notes approach. As others have found, writing up while memories are fresh is beneficial [25], however this may need to be balanced with ensuring feasible fieldwork logistics.

Researchers that plan to use the fair note method must factor training and timely supervision into timelines and staff costs, as it can take a week of training and up to two weeks of intensive supervision for data collectors to become proficient with the method. Using the fair note approach, allowed the team to iterate steps and findings during data collection and think reflectively and analytically. Interviewers reported that writing fair notes improved their interviewing skills. Although the data were rich and relevant, it may be that the completeness and accuracy of the data are low.

Using audio recording to expand field notes allowed verbatim quotes to be added and the completeness of write-ups to be checked, and is recommended [9]. However, we found that interviewers relied on the tape recordings, and that this increased the write-up time and decreased reflection and analytical thinking. There is no consensus on how audio recording should be used to write fair notes [5, 9]; our experience supports listening to the audio recording after the field notes have been expanded from memory, but this may face resistance from interviewers as the existence of the tape made the fieldworkers reluctant to rely on their memories.

## Data Availability

N/A.
